# Combating drug resistance in acute myeloid leukaemia by drug rotations: the effects of quizartinib and pexidartinib

**DOI:** 10.1186/s12935-021-01856-5

**Published:** 2021-04-08

**Authors:** Jingmei Yang, H. Jonathan G. Lindström, Ran Friedman

**Affiliations:** 1 Department of Chemistry and Biomedical Science, Linnaeus University, Kalmar Campus, Kalmar, 391 82 Sweden; 2 Present Address: Faeth Therapeutics Inc., 237 Kearny Street, #9245, San Francisco, CA 94108 US

**Keywords:** Acute myeloid leukaemia, FLT3, kinase inhibitors

## Abstract

**Background:**

Acute myeloid leukaemia (AML) is an aggressive blood cancer. In approximately 30% of the cases, driver mutations in the FLT3 gene are identified. FLT3 inhibitors are used in treatment of such patients together with cytotoxic drugs or (in refractory AML) as single agents. Unfortunately, resistance to FLT3 inhibitors limits their efficacy. Resistance is often due to secondary mutations in the gene encoding the molecular target. The gatekeeper mutation F691L confers resistance to specific FLT3 inhibitors such as quizartinib, but pexidartinib is much less resistance to this mutation. Pexidartinib alone is however sensitive to many other resistance mutations. In chronic myeloid leukaemia (CML), it has been suggested that rotation between drugs with a different landscape of resistance mutations might postpone the emergence of resistance.

**Methods:**

We studied the effect of quizartinib and pexidartinib in AML cell lines that express FLT3 (MOLM-14 and MV4-11). Using a rotation protocol, we further examined whether the emergence of resistance could be postponed. Computational modelling was used to analyse the onset of resistance and suggest which mutations are most likely to occur in a quantitative fashion.

**Results:**

The cells were sensitive to both inhibitors but quickly developed resistance that could be inherited, suggesting a genetic origin. Rotation protocols were not useful to postpone the emergence of resistance, which implies that such protocols, or changing from pexidartinib to quizartinib (or vice-versa) should not be used in patients. The computational modelling led to similar conclusions and suggested that F691L is the most common mutation to occur with quizartinib, and also when both drugs are used in rotation.

**Conclusions:**

AML patients are not likely to benefit from a quizartinib/pexidartinib rotation protocol. A combination of tyrosine kinase inhibitors (with different molecular targets) might be more useful in the future. Development of specific FLT3 inhibitors that are less sensitive to resistance mutations might also lead to a better outcome.

## Background

FMS-like tyrosine kinase 3 (FLT3) is a class III receptor tyrosine kinase. Normally involved in haematopoietic stem cell development, the protein also plays a significant role in acute myeloid leukaemia (AML). Activating mutations in the gene encoding FLT3, and overexpression of FLT3, are commonly associated with AML. These mutations often involve duplications that occur at (rarely) or upstream of (more often) the kinase domain (internal tandem duplications, ITDs [1, 2]). Such insertions increase the flexibility of the protein’s hinge domain, which in turn drives the mutated kinase towards an active state [3, 4]. Inhibition of FLT3 is one of the approaches that are considered in treating the disease in such cases [5]. Indeed, three FLT3 inhibitors were approved for AML treatment: midostaurin (Rydapt^®^), gilteritinib (Xospata^®^) and quizartinib (Vanflyta^®^, approved in Japan).

The paradigm of targeted cancer therapy calls for the use of highly specific drugs that maximise therapeutic benefits and minimise the risk for toxicity. This works well in cancers such as chronic myeloid leukaemia (CML), where a single molecular target is decisive for tumour proliferation [6]. The situation appears to be more complex in AML, where drug combinations are suggested to be used even when FLT3 is overexpressed and mutated [7]. The FLT3 inhibitor midostaurin is widely used as a drug in FLT3-mutated AML. In spite of its capability to inhibit multiple targets, midostaurin is not used as a single-agent but together with chemotherapy. Apparently, midostaurin is not efficient enough in itself due to binding to plasma proteins [8], and its usefulness is due to inhibiting multiple kinases. Multiple kinase inhibitors act on multiple targets, which might be an asset as such inhibitors can be more effective. Unfortunately, this often comes with the associated cost of more severe side effects since even house-keeping kinases are affected. To make things even more complex, some cellular proteins may even have a protective role against the tumour; such proteins should of course not be inhibited [9].

Gilteritinib is a kinase inhibitor with a narrower range of targets, and is used as monotherapy for refractory FLT3-mutated AML [7]. Quizartinib has been developed as a specific FLT3 inhibitor and was found to be highly effective [10]. Unfortunately, resistance is quickly developed in AML patients when treated with quizartinib, often due to secondary mutations in the gene encoding FLT3 [11]. Pexidartinib (Turalio^®^) has shown efficacy against FLT3-ITD carrying the F691L resistance mutation, but other secondary mutations make the tumour resistant to pexidartinib as well [12]. Biophysical and computational studies could explain the reason behind resistance to quizartinib and pexidartinib [13–16] and suggest new means of inhibition [17]. Furthermore, efforts are being made to develop additional FLT3 inhibitors [18]. One of the interesting ideas in this respect is the development of dual-specificity inhibitors that target not only FLT3 but also other molecular targets [19]. However, in this case there is a risk for toxicity and even reduced efficacy if biological mechanisms that act against the tumour are inhibited. It may be possible to tune the use of multiple inhibitors based on biomarkers [20–22], while also considering pharmacokinetics [23]. However, this requires precise knowledge on the expression of multiple targets and the degree of their inhibition by drugs, which is not commonly available.

Recently, it has been suggested that the emergence of drug resistance could be postponed by use of drug rotation rather than drug combination. The underlying idea is that by using two drugs that have a high affinity to the same target but non-overlapping resistance mutation profile (i.e., there are resistant mutations that are unique to each drug), it should be possible to postpone the emergence of resistance that would manifest in the clinic. A knowledge-based computational study suggested that the right combination of drugs and rotation times would indeed be useful to this aim in CML [6, 24]. Here, we examine whether a rotation between quizartinib and pexidartinib might be useful for postponing the emergence of resistance in AML, using two AML cell lines that express FLT3-ITD (MOLM-14 and MV4-11). Knowledge based computer simulations are used to interpret the results and survey the mutational landscape in the tumour.

## Materials and methods

### Reagents

Quizartinib was purchased from AdipoGen Life Sciences. Pexidartinib was purchased from Selleck Chemicals LLC. MTS, (3-(4,5-dimethylthiazol-2-yl)-5-(3-carboxymethoxyphenyl)-2-(4-sulfophenyl)-2H-tetrazolium) was purchased from Promega. Dimethyl sulfoxide (DMSO), fetal bovine serum (FBS), Gibco cell culture media (RPMI-1640 and IMDM) and antibibiotics (1% penicillin-streptomycin, Pen-Strep) were bought from Thermo Fisher Scientific.

### Cell cultures

MV4-11 and MOLM-14 were a generous gift from Prof. Stefan Fröhling, National Center for Tumor Diseases, Heidelberg, Germany. MV4-11 was maintained in 10% FBS and 1% Pen-Strep in IMDM while MOLM-14 was maintained in 10% FBS and 1% Pen-Strep in RPMI-1640. Cells were maintained at $0.5 \times 10^{6}$ to $1.5 \times 10^{6}$ cells/mL and split saturated culture every 2 to 3 days by diluting for 2 to 3 times. Incubation was performed at T = 37$^{\circ }$C and 5% CO$_2$.

### Cell viability assays

Exponentially growing cells were seeded at a density of 50000 (MV4-11) or 30000 (MOLM-14) cells per well in a 96-well plate and treated with DMSO (control) or a dose titration of inhibitors (pexidartinib and quizartinib). MTS was added to the cells 48 hours following the addition of inhibitors. In living mammalian cells, MTS is converted to a formazan compound that has an absorbance maximum at 490 nm. The resulting signals at 490 nm were measured after two hours using a fluorescence plate reader. Viability was calculated as a percentage of cells treated with DMSO. Numerical IC50 values were calculated with non-linear best-fit regression analysis using the Prism 5 software. The model was:

1 $$\begin{aligned} f(x) = max - \frac{max-min}{1+\left( \frac{x}{IC50}\right) ^\alpha } \end{aligned}$$

where *x* is the concentration of the drug, *f*(*x*) is the measurement, *max* and *min* are the maximum and minimum values that are measured, and $\alpha$ is the Hill slope.

The MTS assay was performed according to the supplier’s recommended protocol. The dye was kept frozen and thawed by leaving it at room temperature for 90 min. 20 $\upmu$L of MTS solution was added to each well and left for incubation in the dark for 1–4 h, before measuring the absorbance at 490 nm.

### Drug rotations in MOLM-14 and MV4-11 cell lines

To study the effect of inhibitors on MOLM-14 and MV4-11 cells, these cell lines were grown in the presence of quizartinib and pexidartinib at a concentration that matched the IC50 value for the drugs (quizartinib: 0.384 nM for MOLM-14, 0.313 nM for MV4-11; pexidartinib: 51.4 nM for MOLM-14, 37.9 nM for MV4-11). In addition, we studied the effect of a higher concentration of pexidartinib (163.1 nM for MOLM-14 and 530.2 nM for MV4-11, corresponding to the IC90 values) on the cells. To differentiate between the two treatments, i.e., between pexidartinib (IC50) and pexidartinib (IC90) we refer to the latter as pexidartinib*. Of note, a phase I/II clinical study pointed out that achieving such degree of inhibition in patients is possible with pexidartinib [25].

Adaptation experiments were run in 24-well plates, which were set up as shown in Table 1. Each well was set up with $10^5$ cells in 1 mL medium with the appropriate inhibitor. The drugs were added once, at the start of each 6-day period, to which we refer as “generation”. The number of cells was counted every two days. After 6 days, the medium in all wells was replaced with fresh medium; in the control wells, the inhibitors were unchanged, and in the rotation wells, the inhibitors were changed as shown in Table 1. At the same time, the culture was diluted such that, at the start of each generation, each well contained $10^5$ cells in 1 mL medium. Each set of cells was grown in duplicate separate wells, and sampling was performed twice for each well, yielding four points of data for each measurement. Growth rates were calculated at day 3 and day 6 using the ratrack tool [26], https://github.com/Sandalmoth/ratrack.

**Table 1:** Schedule for treatment-rotation experiments

Generation	Treatment					
1	Pexidartinib	Pexidartinib*	Quizartinib	Pexidartinib*	Quizartinib	Pexidartinib*
2	Pexidartinib	Pexidartinib*	Quizartinib	Quizartinib	Pexidartinib*	Quizartinib
3	Pexidartinib	Pexidartinib*	Quizartinib	Pexidartinib*	Quizartinib	Quizartinib
4	Pexidartinib	Pexidartinib*	Quizartinib	Quizartinib	Pexidartinib*	Pexidartinib*
5	Pexidartinib	Pexidartinib*	Quizartinib	Pexidartinib*	Quizartinib	Quizartinib
6	Pexidartinib	Pexidartinib*	Quizartinib	Quizartinib	Pexidartinib*	Quizartinib
Figures	2	3	4	5	6	7

Six treatments were performed, three controls (no rotations) and three rotation experiments where drugs were replaced according to the generations. The three columns to the left represent controls. The cells were treated by quizartinib at IC50, or pexidartinib in two concentrations, one that matches the IC50 and one that matches the IC90 (indicated as Pexidartinib*). The three columns to the right represent the actual rotation experiments in different sequence. Cells were washed after each generation (t = 6 days) to remove any trace of the drug, even for the control arm. Figure numbers refer to the figures showing the results

A Luna-II Automated Cell Counter was used together with counting slides manufactured by Logos Biosystems for all cell counts. The cell counting procedure was as follows. First, the culture medium in the well was gently mixed. Thereafter, 10 μL of culture medium was extracted from the well, and mixed with 10 μL trypan blue in a 1.5 mL Eppendorf tube. After mixing the medium and trypan blue, 10 μL of the mixture was deposited onto a disposable cell counting slide, which was used in the cell counter.

### Simulations

Simulations of cell population developing resistance were run using the wollsey package [6], which is freely available for download at https://github.com/Sandalmoth/wollsey-public. The protocol was the same as in [6]. Briefly, given a starting population of cells that do not carry any resistance mutations and a table listing mutations and their IC50 values, the development of mutations under treatment is followed stochastically. A treatment protocol is also included. Here, the protocol matched the experiments (treatment by either of the drugs alone or rotations thereof).

Given that we model an active form of cancer, the number of cells in the simulation was kept roughly constant at $1.0 \times 10^6$ cells, which enables to follow on the development of resistance. The probability for mutation was set to $1.0 \times 10^{-7}$ per base pair, consistent with estimations in blood cancers and about two order of magnitude larger than the mutation rate in normal cells. The simulations were run $1.0 \times 10^5$ times to ensure sufficient statistics. The growth rate was set to once per day and the death rate to 1/10 of the growth rate (there is a spring parameter that ensures that the population of cells do not overgrow). Relative IC50 values per mutation are given in Table 2. Rotation between the drugs was set to have the same period as in the experiments (6 days).

**Table 2:** Fold-IC50 values for FLT3 mutations relative to FLT3-ITD

Mutation	Fold IC50 quizartinib	Fold IC50 pexidartinib
None	1	1
F691L	329	3
D835A	10	18
D835E	6	9
D835F	1474	415
D835G	10	13
D835H	45	40
D835I	718	1937
D835N	7	10
D835V	563	320
D835Y	183	206
D835Del	320	121
D839G	6	27
Y842C	106	48
Y842H	58	49

This is the data used in simulations of the cells. The values were calculated based on Refs. [12, 35, 36]

## Results

### Quizartinib and pexidartinib reduce the viability of AML cells in a concentration-consistent manner

**Fig. 1:**
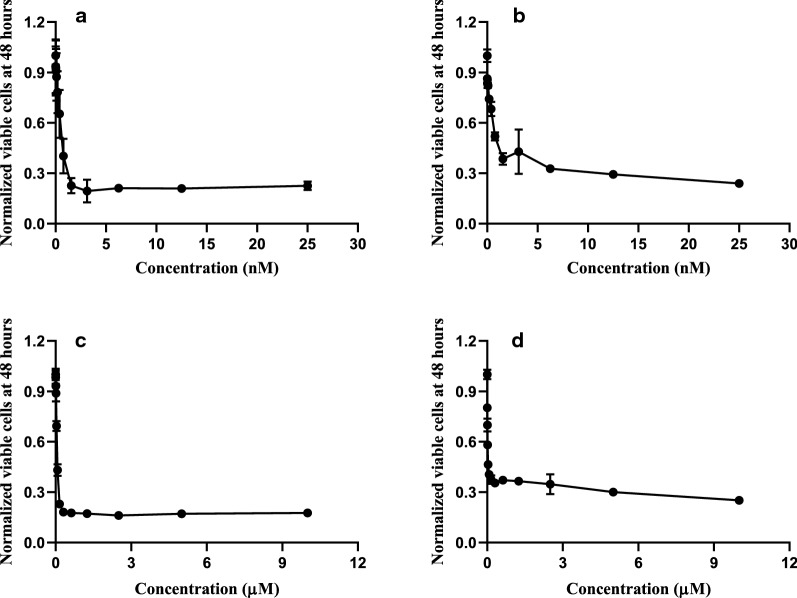
Quizartinib and pexidartinib are effective inhibitors of FLT-dependent AML cell lines. The viabilities (normalised with respect to control, i.e., 0.25% DMSO) of MOLM-14 (**a**, **c**) and MV4-11 (**b**, **d**) are shown as the cells are treated by increasing concentrations of quizartinib (**a**, **b**) and pexidartinib (**c**, **d**). Each measurement was carried out in triplicate after 48 h of growth in presence of an inhibitor in a given concentration. Cell viabilities are estimated from an MTS assay (see “Methods” section for details)

Prior to testing the development of drug resistance in AML cell lines we verified that the cell lines at hand were sensitive to the drugs. Indeed, incubation of MOLM-14 and MV4-11 cells with quizartinib and pexidartinib for 48 h revealed that the number of viable cells was reduced in a concentration-consistent manner (Fig. 1). Consistent with previous studies [12, 27, 28], we found quizartinib and pexidartinib to have half maximally inhibitory coefficients (IC50) in the sub-nanomolar and low-nanomolar range, respectively (Table 3).

**Table 3:** Inhibitory coefficients of pexidartinib and quizartinib

	IC50		IC90	
	MOLM-14	MV4-11	MOLM-14	MV4-11
Quizartinib	0.38 ± 0.17	0.31 ± 0.16		
Pexidartinib	51 ± 4	37 ± 6	160 ± 27	530 ± 48

Values are averages (± standard deviations) in nM

### AML cells quickly develop resistance to quizartinib and pexidartinib

After establishing the sensitivity of the cells to the two inhibitors, we examined whether the cells could develop resistance to the drugs, and whether washing and re-introducing the drug modified the resistance profiles. To this aim, we incubated the cells with quizartinib and pexidartinib and followed on their growth for 6 days, after which the cells were washed, removing any of the drug before the cells were treated again. Six cycles (generations) of grow-treat-wash were performed, and the growth rate was estimated at days 3 and 6. Washing was necessary to avoid exposing the cells to the two drugs at the same time, since metabolism of the drugs in these cells is limited.

**Fig. 2:**
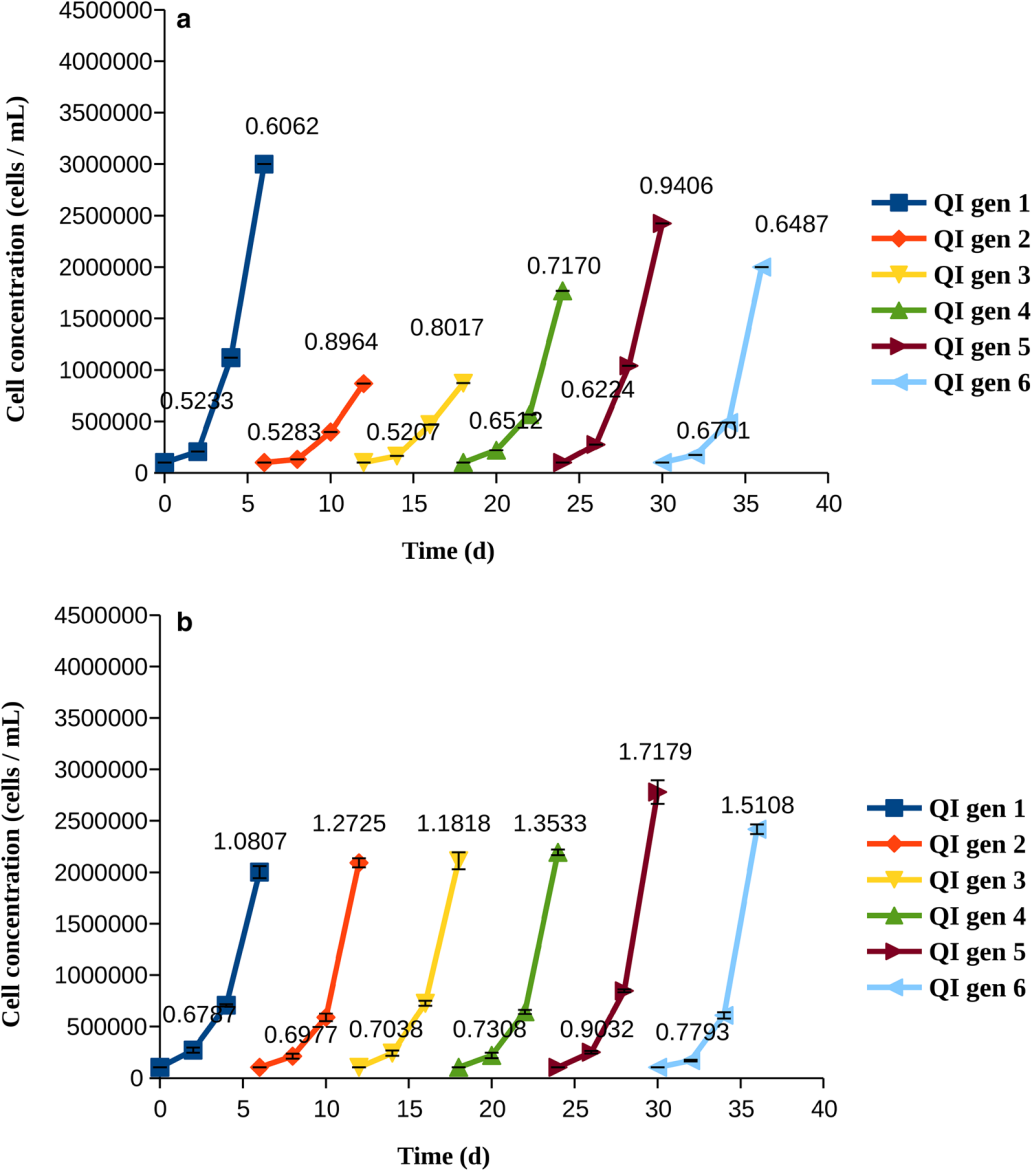
Cell counts and growth rates for cells treated with quizartinib at IC50 (QI). The growth rates (divisions per cell and day), were calculated at days 3 and 6 and are indicated on each line. **a** MOLM-14 cells, **b** MV4-11 cells

**Fig. 3:**
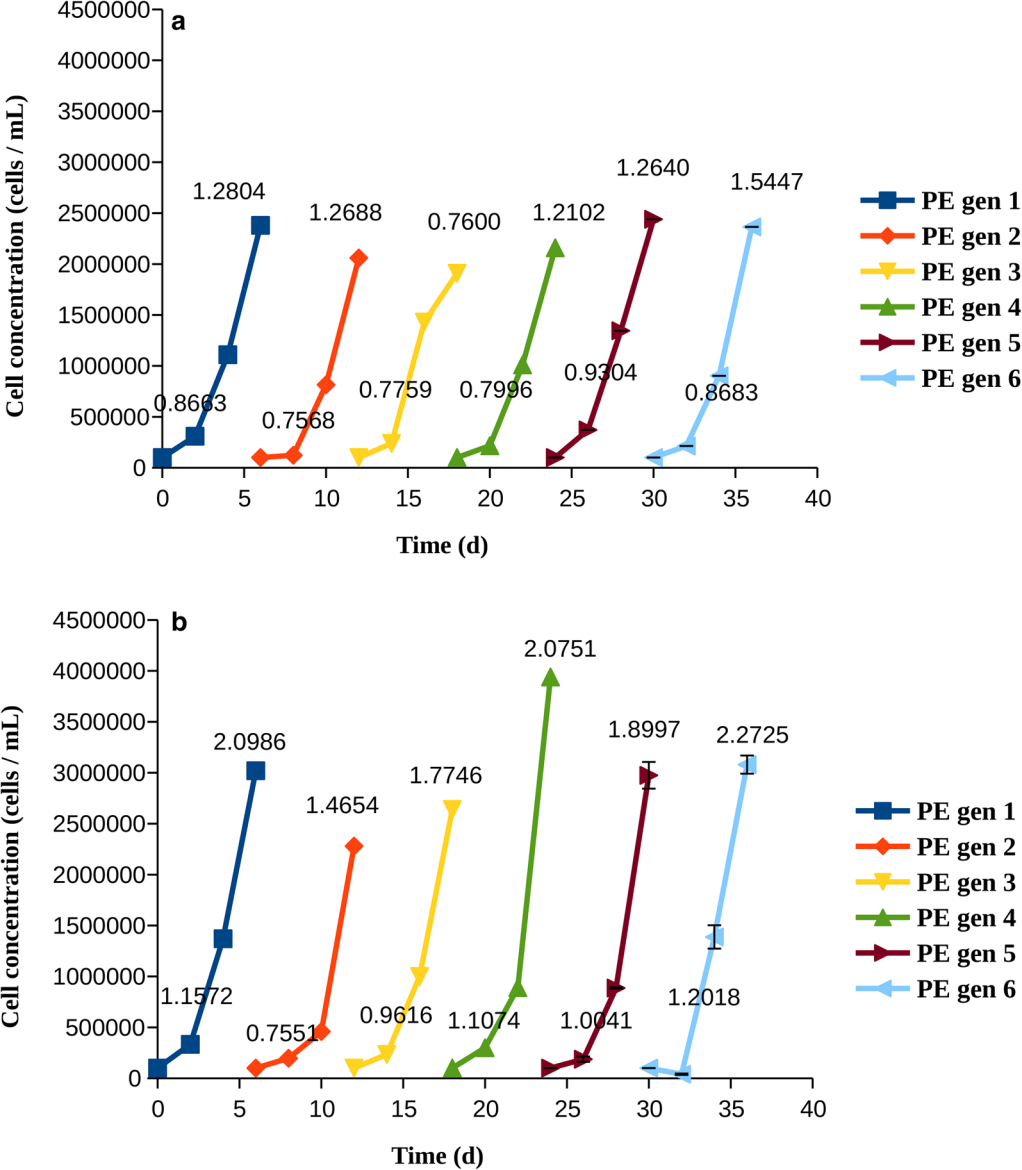
Cell counts and growth rates for cells treated with pexidartinib at IC50 (PE). The growth rates (divisions per cell and day), were calculated at days 3 and 6 and are indicated on each line. **a** MOLM-14 cells **b** MV4-11 cells

The growth of cells subject to treatment with quizartinib and pexidartinib at a concentration that is equal to their IC50 is shown in Figs. 2, 3, respectively. With both drugs, the cells grew slowly at the first days, but then became resistant, as evidenced by comparison of the number of cells and growth rates at days 3 and 6 (of note, a smaller growth rate at day six might indicate that the cell density becomes too high, which in itself limits the growth). None of the cell populations were impaired by the washing procedure, as shown by the continued growth at subsequent generations without slowing with respect to generation 1. When MV4-11 cells were treated by quizartinib, there was a gradual increase in the number of cells at day 6 upon subsequent cycles (compare generations 1–3 to generations 4–6 in both panels of Fig. 2b). The number of cells was surprisingly high at day 6 of generation 1 when MOLM-14 cells were treated with quizartinib. While we are not able to know the exact reason for this, we note that other mechanisms than resistance mutations may influence the growth [9, 22, 29], but such mechanisms are often not persistent. We note that the number of cells measured on day six is also higher than the corresponding number when treated by pexidartinib (generation 1, day 6).

**Fig. 4:**
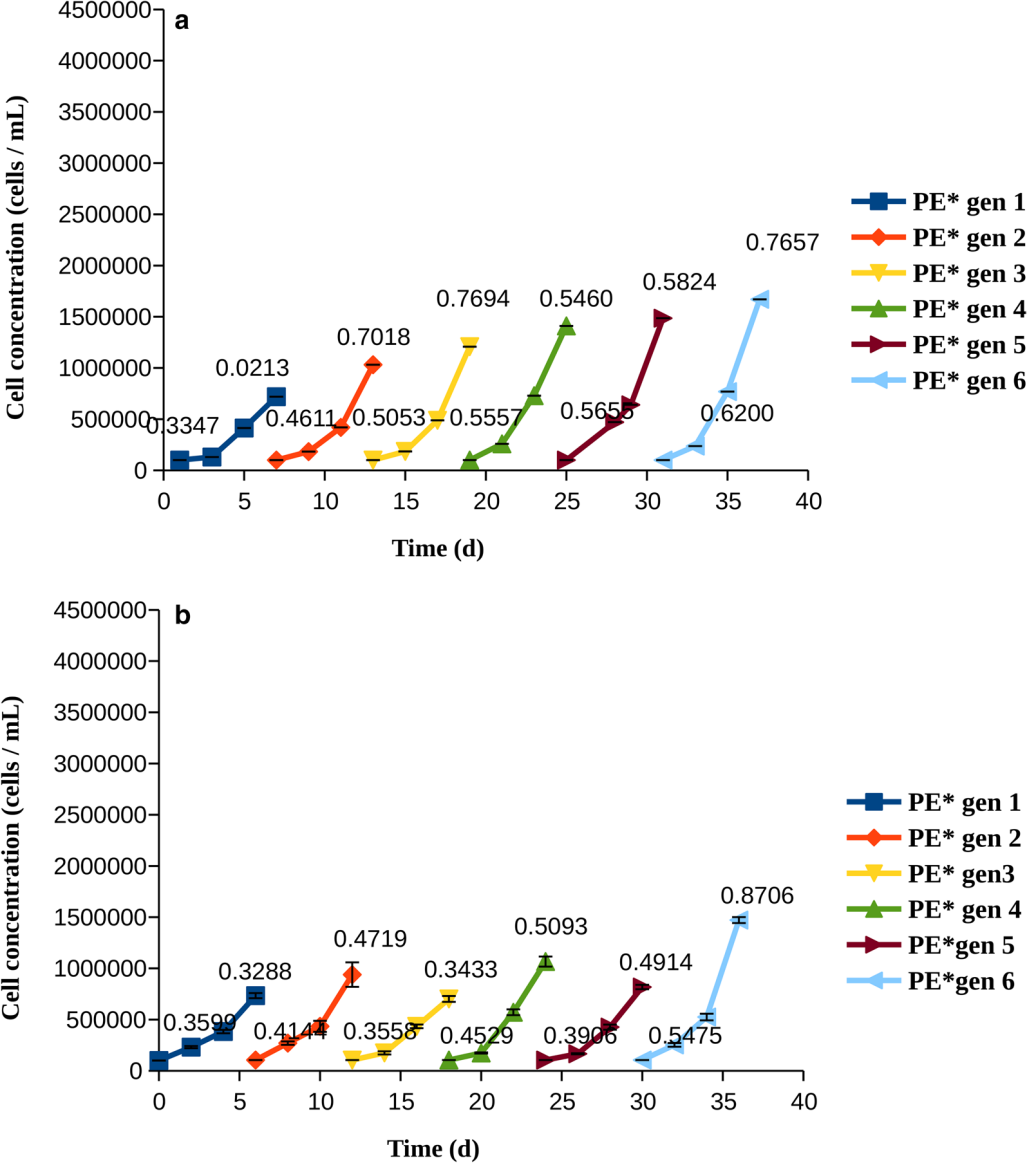
Cell counts and growth rates for cells treated with pexidartinib at IC90 (PE*). The growth rates (divisions per cell and day), were calculated at days 3 and 6 and are indicated on each line. **a** MOLM-14 cells, **b** MV4-11 cells

Resistance was more apparent in pexidartinib than in quizartinib, as shown by higher growth rates in both cell lines. Accordingly, we examined whether cells treated with pexidartinib could tolerate a higher concentration of the drug, and whether this would reduce the growth in the long run. Indeed, cells developed resistance more slowly when pexidartinib was used at a higher concentration (Fig. 4). In MOLM-14 cells, it could clearly be shown that the cells had become more resistant to therapy at each cycle, as indicated by higher proliferation and growth rates at day 3 (Fig. 4a). In MV4-11 cells, the situation was less clear-cut, although in general there was an increase in both the growth rate and the cell number with the generation; both reached a maximum in generation 6 (Fig. 4b).

### Rotating between quizartinib and pexidartinib selects for adaptations that generate resistance to both

Given that (1) resistance was observed with both drugs, and became more evident with each generation (2) resistance was previously shown to depend on resistance mutations, and (3) the mutational landscape is different between the two drugs, we set to examine whether a rotation protocol, where we periodically replace between the drugs could postpone the emergence of drug resistance. If cells that become resistant to one drug are not resistant to the other, such cells will be wiped out upon modifying the drug; this would result in delaying the onset of an overall treatment-defiant tumour. On the other hand, there are mutations that lead to resistance against both drugs. Combination therapy would select for such mutations whereas a cleverly chosen rotation protocol can result in the cells staying sensitive to therapy for a longer time [6].

**Fig. 5:**
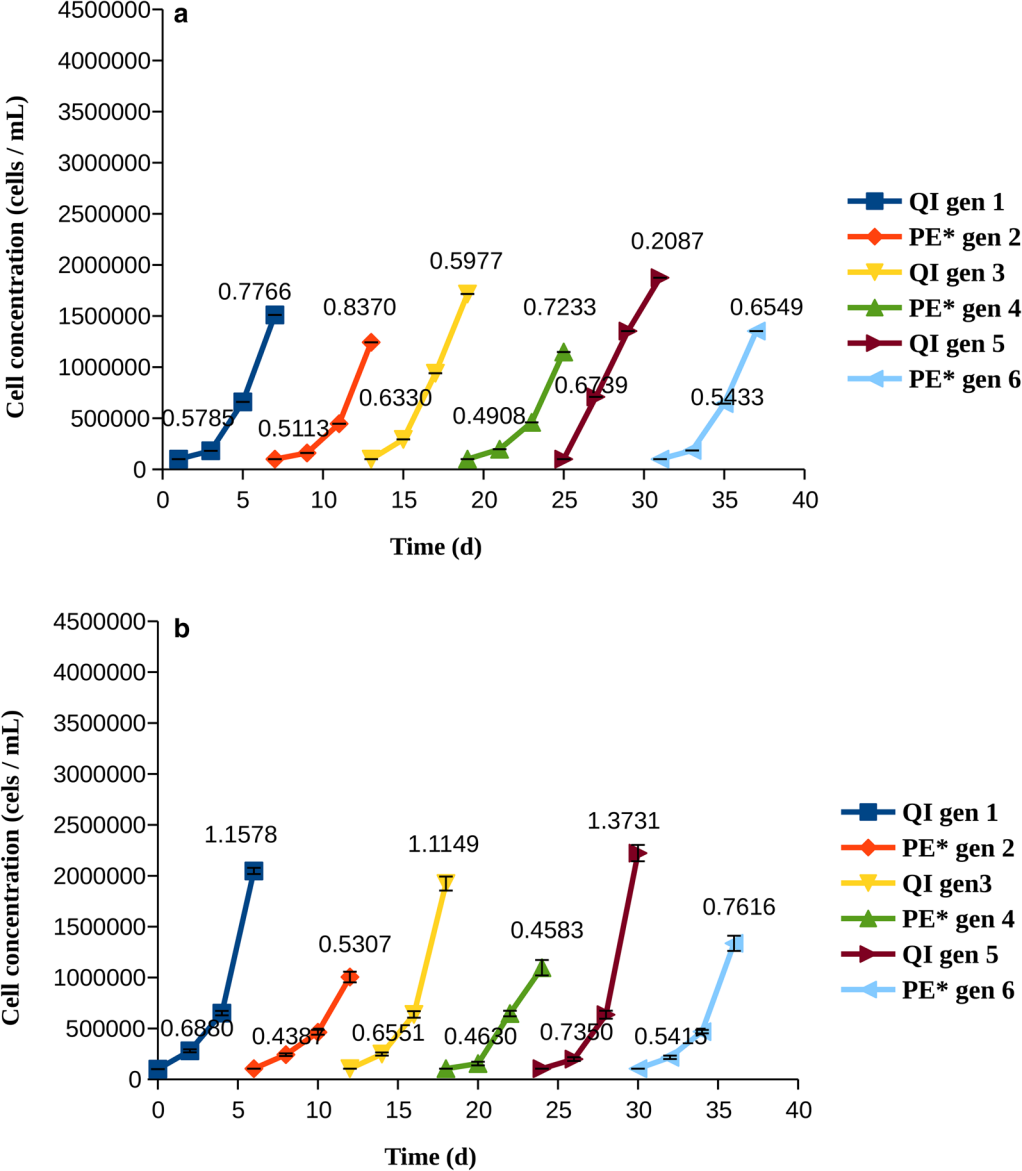
Cell counts and growth rates for cells treated with quizartinib at IC50 (QI) at generations 1, 3, and 5 pexidartinib at IC90 (PE*) at generations 2, 4, and 6. The growth rates (divisions per cell and day), were calculated at days 3 and 6 and are indicated on each line. **a** MOLM-14 cells, **b** MV4-11 cells

**Fig. 6:**
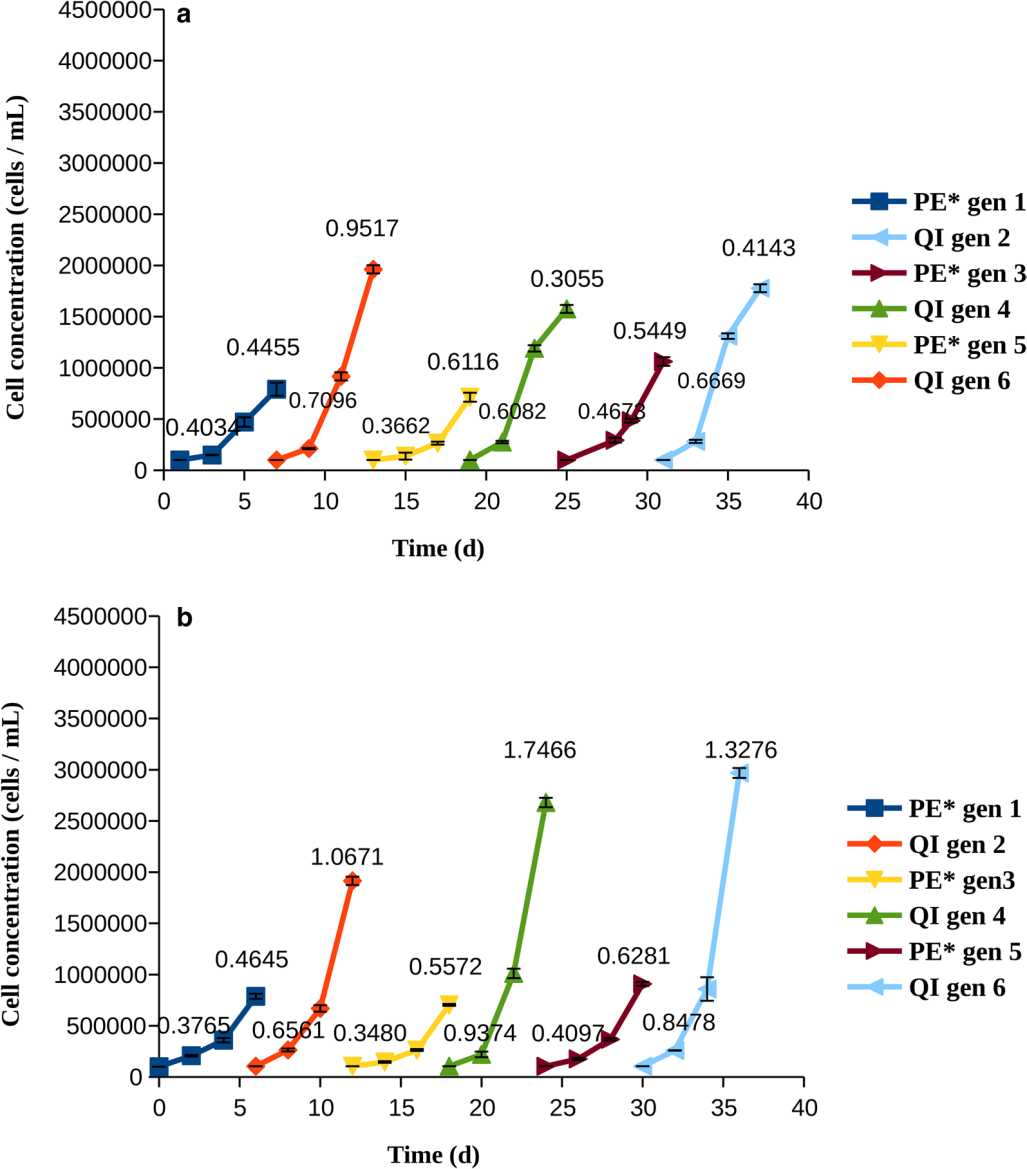
Cell counts and growth rates for cells treated with pexidartinib at IC90 (PE*) at generations 1, 3, and 5 and quizartinib at IC50 (QI) at generations 2, 4, and 6. The growth rates (divisions per cell and day), were calculated at days 3 and 6, and are indicated on each line. **a** MOLM-14 cells, **b** MV4-11 cells

Given that resistance occurred especially quickly with pexidartinib at IC50, we applied a rotation protocol between quizartinib at IC50 and pexidartinib at IC90. The aim was to study the cells when resistance does develop, but not too fast. The growth of the cells with 1:1 quizartinib:pexidartinib and 1:1 pexidartinib:quizartinib rotation protocols is shown in Figs. 5 and 6, respectively.

**Fig. 7:**
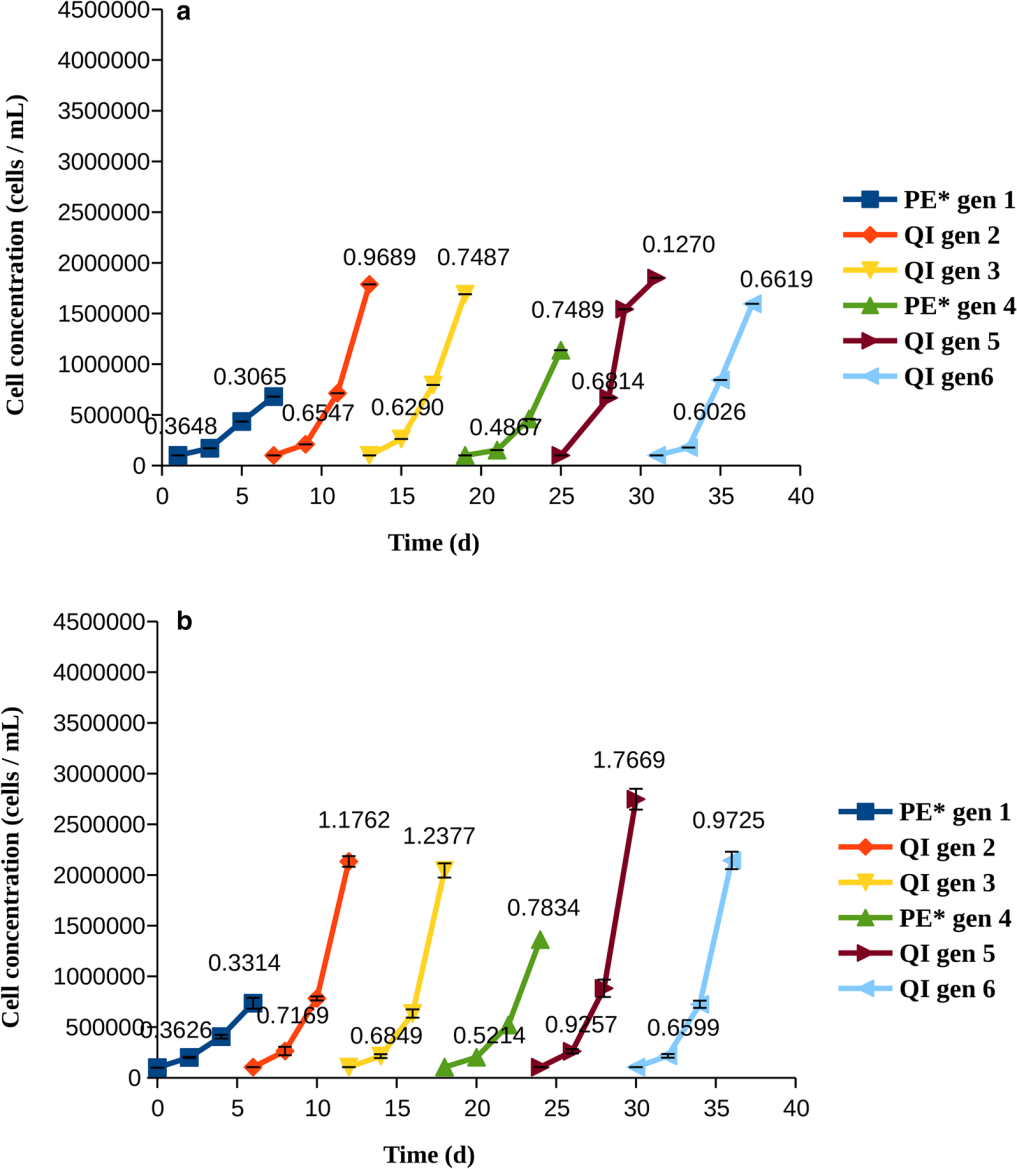
Cell counts and growth rates for cells treated with pexidartinib at IC90 (PE*) at generations 1 and 4 and quizartinib at IC50 (QI) at generations 2, 3, 5, and 6. The growth rates (divisions per cell and day), were calculated at days 3 and 6, and are indicated on each line. **a** MOLM-14 cells, **b** MV4-11 cells

The results of these rotation experiments do not suggest that such a protocol could be useful in AML. Regardless of the drug which had been used first in the rotation, resistance was observed in all cases and did not slow down upon switching between drugs. Interestingly, a clear increase of growth with generation was observed only for the pexidartinib:quizartinib protocol in MV4-11 cells. As the cells did not seem to become more tolerant to pexidartinib after treatment with quizartinib, we further tried a protocol where quizartinib was used for two consecutive cycles. These experiments were carried out to examine if such treatment selected mostly for quizartinib-resistant mutations, or whether long treatment with quizartinib was likely to make the cells resistant to pexidartinib as well. The results (Fig. 7) revealed that two consecutive generations treated with quizartinib made the cells less sensitive to pexidartinib. It is thus clear that pexidartinib is unlikely to benefit patients that were treated with quizartinib before.

### The mutational landscape is complex, with F691L often the most common resistance mutation

Given the multiple mutations that can confer resistance to quizartinib and pexidartinib, analysis of the mutational landscape is complex and calls for computer-aided methods, as it is not realistic to follow on the emergence of more than few resistance mutations from sampling and sequencing. We have developed a method to simulate the evolution of resistance mutations in cancers based on the growth rate of cells and the degree of inhibition and resistance [6]. Before estimating the mutations, we wanted to see if the pattern of resistance, as predicted by the computational modelling, agrees with the experimental results.

To estimate the establishment of resistance, we define WT$_{1/2}$ as the time that would take a tumour to include a cell population where 50% of the cells carry resistance mutations (here “wild type” refers to the initial state of the molecular drug target, which in FLT3 includes an ITD activating mutation). The longer it takes the tumour to reach WT$_{1/2}$ population, the more sensitive it is to therapy.

**Fig. 8:**
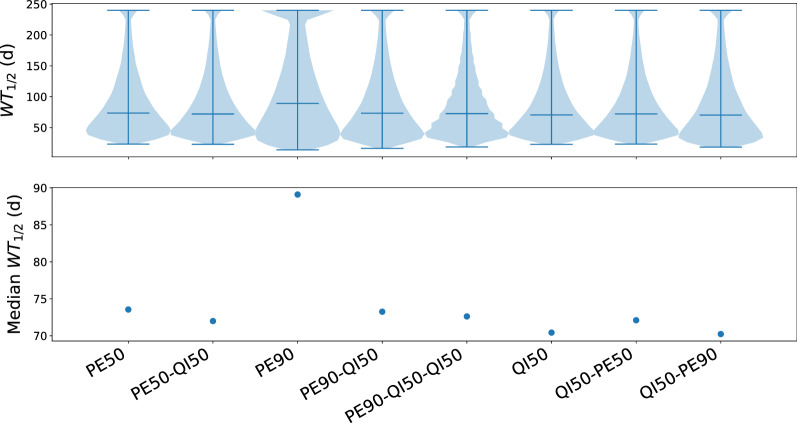
Simulations of treatment corresponding to the rotation protocols. The top panel is a violin plot showing the probability densities of WT$_{1/2}$ values from the simulations (Y-axis: WT$_{1/2}$, X-axis: probability densities for each protocol). The horizontal lines show minimum, median and maximum values. The bottom panel shows the median WT$_{1/2}$. Higher WT$_{1/2}$ values implicate that the tumour is sensitive to the treatment for a longer time. The figure was obtained by simulations of the mutational landscape using the wollsey stochastic evolution simulator [6]

The distributions and median times to reach WT$_{1/2}$ for are shown in Fig. 8. Examination of the median WT$_{1/2}$ values indicates that high concentration of pexidartinib was the most efficient treatment of all six monotherapies or combinations that we tested in terms of limiting growth resistance, and that all other protocols are roughly equal in this sense. This finding is in agreement with the experimental results (Figs. 2, 3, 4, 5, 6, 7).

Whereas treatment with high concentration of pexidartinib seems to be the best strategy of those we applied based on cell growth and development of resistance, the lower end of the probability distributions were wide, which reveals that resistance is likely to develop very quickly in some patients, even those treated by high concentration of pexidartinib (top panel of Fig. 8). Overall, the simulations suggest that resistance to therapy by FLT3 inhibitors would develop within few months, irrespective of the treatment protocol. This time frame is consistent with clinical studies involving quizartinib [30, 31].

**Fig. 9:**
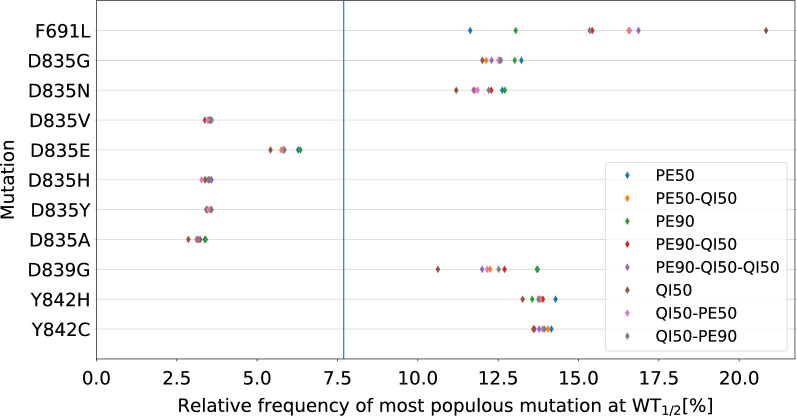
Distribution of most common mutation at WT$_{1/2}$ for FLT3$^+$-AML. The relative frequency of each mutation is given for each treatment protocol. The figure was obtained by simulations of the mutational landscape using the wollsey stochastic evolution simulator [6]. The blue line indicates the expected mutation frequency if all mutations were equally likely and equally fit

After verifying that the computational model reproduced the resistance pattern observed in the cells, we went on to analyse the prevalence of mutations (Fig. 9). In all cases, a combination of mutations was observed when the WT$_{1/2}$ point had been reached. The gatekeeper mutation F691L was the most common mutation in all protocols involving quizartinib; it was rather frequent even with pexidartinib alone. Pexidartinib was developed to overcome this mutation, but is less effective for cells that carry it compared to FLT-ITD without additional resistance mutations. Other mutations that were common are D835N/G (more often with pexidartinib or rotations with pexidartinib), D839G (with pexidartinib, and decreasing in frequency when rotations are used), and Y842H/C.

## Discussion

The aim of this study was to examine whether rotating between two drugs with a different (yet partially overlapping) mutational profile could postpone the emergence of resistance in FLT3$^+$-AML. To this end, we developed and applied a rotation protocol in AML cell lines, whereby drugs are periodically switched. Postponing the emergence of drug resistance is highly desired in many cancers, and is a matter of urgency in AML. Aggressive chemotherapy (cytarabine and either daunorubicin or idarubicin) is the current standard of care for AML, often together with the kinase inhibitor midostaurin for patients that carry activating FLT3 mutations [32]. Unfortunately, whereas AML is more likely to occur at older age, older patients are at higher risk when undergoing such intensive treatment. In addition, the cancer may become refractory to chemotherapy, leaving FLT3-inhibitors such as gilteritinib or quizartinib the only treatment option [8]. Development of resistance mutations is therefore disastrous.

Examination of total growth and growth rates revealed that AML cells quickly became resistant to quizartinib and pexidartinib, as shown by their growth rates increasing with time (Figs. 2, 3). Resistance to pexidartinib was particularly fast to develop. The process was slower with a higher concentration of pexidartinib, but the cells gradually overcame the drug and their growth rates were increased. Resistance to pexidartinib at IC90 was clearly generation-dependent, indicating that mutants that confer resistance became increasingly more common in the cell population.

From an evolutionary standpoint, treating a tumour leads to very strong impairment of its fitness [33, 34], as cells are not able to divide and the population shrinks. Mutations that provide resistance to the drug suddenly gain a large benefit when it comes to adaptation. When considering kinase inhibitors, gate-keeper mutations, that involve an amino acid residue at the nucleotide binding site of a kinase are observed quite often [29]. Examples include T790M in EGFR and T315I in Abl1. The corresponding mutation in FLT3 is F691L. This common resistance mutation makes the tumour insensitive to quizartinib, whereas treatment with pexidartinib might still be effective. The presence of mutations that confer resistance only to one drug but not to the other suggests that both treatment options could be considered. Whereas it is counter-productive to treat patients with two drugs that target the same molecular target in the same way, a study of CML therapy suggested that rotating between two drugs might be effective to postpone resistance [6]. Following our study, this approach is not recommended for treating AML as none of the rotation protocols performed very well. What is more, longer treatment with quizartinib made the cells more resistance to high concentration of pexidartinib, compared to quizartinib-naïve cells.

The results of a Phase I/II clinical study of pexidartinib have recently been reported [25]. As a single agent, pexidartinib was shown to be less effective than quizartinib or gilteritinib and the authors suggested that this might be attributed to prior treatment with one of these agents. Our study shows that resistance develops rapidly against pexidartinib and that despite the relative potency of pexidartinib against FLT3-ITD/F691L, development of resistance mutations is likely a major reason for relapse. Interestingly, the simulations suggested that it is not a single mutation that is observed but rather a combination thereof (Fig. 9), in all cases. Thus, patients that relapse on quizartinib are unlikely to benefit from pexidartinib.

Encouragingly, simulations of cell growth agreed very well with the experiments. Analysis of the simulations revealed that the cell developed resistance under all conditions and that rotation protocols did not yield any additional benefits. Using a higher concentration of the drug seemed to increase the median WT$_{1/2}$, that is the median time until resistance mutations (of any kind) dominated the population. In principle, this might indicate that aggressive treatment is best even when considering targeted therapies. While this may indeed be the case, the dosage of drugs is often limited by toxicities, so that the clinician is seldom at liberty to choose a higher dose.

Considering the mutations themselves, the simulations suggest F691L to be common even for pexidartinib. This appears to be puzzling as there are more efficient resistance mutations especially against pexidartinib. Of note, the molecular mechanism causing mutations is blind to the effect of these mutations, and any increase in the fold-IC50 may be enough for the mutation to gain a fitness advantage. There are multiple ways to modify the TTT codon for Phe in residue Phe$^{691}$ to Leu, making the mutation likely to occur more often by chance (TTA, TTG and CTT all encode for Leu). In addition, it should be mentioned that there are multiple mutations in residues Asp$^{835}$ that lead to resistance; taken together, mutations in Asp$^{835}$ are more common than F691L.

## Conclusions

Experiments with two AML cell lines confirm the notion that monotherapy with highly specific FLT3 inhibitors alone is subject to resistance. Resistance was more pronounced with each generation, revealing an evolutionary mechanism that likely depends on resistance mutations. Different rotation protocols were not successful in driving the cells towards a treatable state. Simulations of cell growth agreed with the experimental measurements and predicted that the gatekeeper mutation F691L is the most common single resistance mutation to be observed upon using any protocol involving quizartinib, and that it will commonly be observed even under treatment with pexidartinib. Other measures, such as combination therapy may be more promising; for such treatment to succeed it is however necessary to consider different pathways that promote cell proliferation. Finally, given the central role of FLT3 in AML, novel inhibitors that will be more robust to resistance mutation (such that only very few mutations would lead to resistance) may also be of great value. This is since, as the computational modelling shows, when more mutations are possible resistance develops faster.

## Data Availability

Data sharing is not applicable to this article as no datasets were generated or analysed during the current study.

## Abbreviations

AML: Acute myeloid leukaemia
CML: Chronic myeloid leukaemia
FLT3: FMS-like tyrosine kinase 3
ITD: Internal tandem duplication
